# Calcium homeostasis alterations in a mouse model of the Dynamin 2-related centronuclear myopathy

**DOI:** 10.1242/bio.020263

**Published:** 2016-11-15

**Authors:** Bodvaël Fraysse, Pascale Guicheney, Marc Bitoun

**Affiliations:** 1 Atlantic Gene Therapies, INSERM UMR 1089, Université de Nantes, CHU de Nantes, Nantes44200, France; 2 INSERM, UMR_S1166, Sorbonne Universités, UPMC Univ Paris 06, UMR_S1166, Institute of Cardiometabolism and Nutrition (ICAN), Paris75013, France; 3 Research Center for Myology, UPMC Univ Paris 06 and INSERM UMR_S974, CNRS FRE 3617, Institute of Myology, Paris75013, France

**Keywords:** Calcium, Dynamin 2, Centronuclear myopathy, Knock-in mouse model

## Abstract

Autosomal dominant centronuclear myopathy (CNM) is a rare congenital myopathy characterized by centrally located nuclei in muscle fibers. CNM results from mutations in the gene encoding dynamin 2 (DNM2), a large GTPase involved in endocytosis, intracellular membrane trafficking, and cytoskeleton regulation. We developed a knock-in mouse model expressing the most frequent *DNM2*-CNM mutation; i.e. the KI-*Dnm2*^R465W^ model. Heterozygous (HTZ) KI-*Dnm2* mice progressively develop muscle atrophy, impairment of contractile properties, histopathological abnormalities, and elevated cytosolic calcium concentration. Here, we aim at better characterizing the calcium homeostasis impairment in extensor digitorum longus (EDL) and soleus muscles from adult HTZ KI-*Dnm2* mice. We demonstrate abnormal contractile properties and cytosolic Ca^2+^ concentration in EDL but not soleus muscles showing that calcium impairment is correlated with muscle weakness and might be a determinant factor of the spatial muscle involvement. In addition, the elevated cytosolic Ca^2+^ concentration in EDL muscles is associated with an increased sarcolemmal permeability to Ca^2+^ and releasable Ca^2+^ content from the sarcoplasmic reticulum. However, amplitude and kinetics characteristics of the calcium transient appear unchanged. This suggests that calcium defect is probably not a primary cause of decreased force generation by compromised sarcomere shortening but may be involved in long-term deleterious consequences on muscle physiology. Our results highlight the first pathomechanism which may explain the spatial muscle involvement occurring in *DNM2*-related CNM and open the way toward development of a therapeutic approach to normalize calcium content.

## Abstract (precis)

**Summary:** Dynamin 2 mutations cause centronuclear myopathy via unclear mechanisms. We show in a mouse model that changes in cytosolic calcium via incorrect membrane permeability correlate with muscle weakness.

## INTRODUCTION

Centronuclear myopathies (CNM) are rare congenital myopathies characterized by muscle weakness and abnormal centralization of the myonuclei in a large number of muscle fibers in absence of muscle regeneration. Three forms of CNM have been distinguished corresponding to three modes of inheritance (Romero and Bitoun, 2011). The X-linked recessive myotubular myopathy (XLMTM), characterized by severe hypotonia and generalized muscle weakness at birth, is caused by mutations in the *MTM1* gene encoding the myotubularin (Laporte et al., 1996). The autosomal forms of CNM show a wider clinical spectrum from severe neonatal to mild-adult forms (Romero and Bitoun, 2011). The autosomal recessive CNM was linked to mutations in *BIN1* encoding amphiphysin 2 (Nicot et al., 2007), and autosomal dominant CNM (AD-CNM) results from mutations in the *DNM2* gene encoding dynamin 2 (DNM2) (Bitoun et al., 2005). More recently, mutations in *BIN1* have been also associated with AD-CNM (Böhm et al., 2014). No treatment is available for the three forms of CNM and the pathophysiological mechanisms are still largely unknown.

DNM2 is a large GTPase involved in the release of nascent vesicles from biological membranes. DNM2 participates in clathrin-mediated and clathrin-independent endocytosis and in intracellular membrane trafficking. In addition, DNM2 regulates the actin and microtubule networks (Durieux et al., 2010a). The *DNM2* mutations were associated with the entire clinical spectrum encountered in AD-CNM from the classical mild late onset CNM to the more severe neonatal form (Böhm et al., 2012; Romero and Bitoun, 2011). Muscle biopsies from *DNM2*-CNM patients show a characteristic association of three morphological features composed of nuclear centralization, predominance and atrophy of type 1 muscle fibers, and a radial arrangement of sarcoplasmic strands. Additionally, disorganization of the triad, the key structure of the excitation-contraction coupling (ECC) in muscle, has also been reported in some *DNM2*-CNM patients (Cowling et al., 2011; Toussaint et al., 2011) and in mouse muscle over-expressing a DNM2 mutant (Cowling et al., 2011). A similar defect was shown in muscle biopsies from *BIN1*-CNM and *MTM1*-CNM patients (Dowling et al., 2009; Nicot et al., 2007) and *MTM1* knock-down in animal models leads to disorganization of the T-tubule system associated with a reduction in Ca^2+^ release from the sarcoplasmic reticulum (SR) (Al-Qusairi et al., 2009; Dowling et al., 2009).

We have developed a knock-in mouse model expressing the most frequent *DNM2*-CNM mutation; i.e. the KI-*Dnm2*^R465W^ model, which mimics most of the human AD-CNM features (Durieux et al., 2010b). Heterozygous (HTZ) mice progressively develop a muscle phenotype associating impairment of contractile properties, atrophy and morphological abnormalities mainly affecting mitochondria and reticulum. Defective calcium homeostasis has also been evidenced in isolated fibers from flexor digitorum brevis (FDB) muscle from HTZ mice, which exhibit a higher cytosolic calcium concentration (Durieux et al., 2010b). Here, we aim at better characterizing this defect in calcium homeostasis in mechanically dissected fibers of the fast-twitch extensor digitorum longus (EDL) and the slow-twitch soleus muscles from HTZ KI-*Dnm2* mice.

## RESULTS

### Resting cytosolic calcium is higher in EDL from HTZ mice

**Fig. 1.**
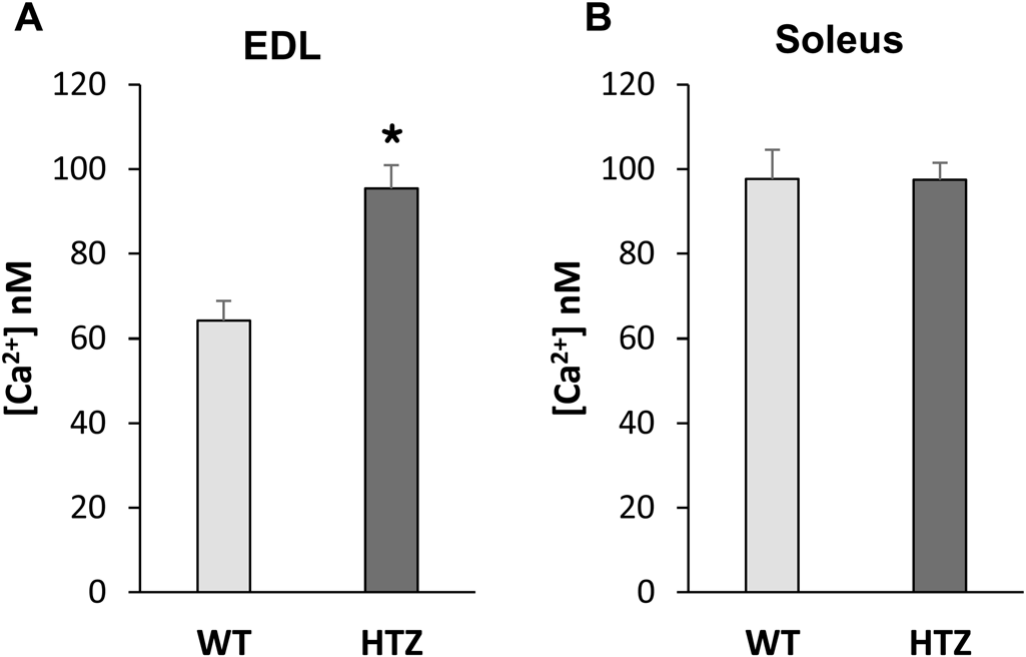
**Resting cytosolic calcium concentration in WT and HTZ EDL and soleus muscles.** [Ca^2+^]_c_ was measured in Fura-2-loaded muscle fiber bundles. The bars represent mean [Ca^2+^]_c_±s.e.m. calculated in *n* cells from *N* animals (*n*/*N*: 85/5 for WT EDL, 131/6 for HTZ EDL, 118/5 for WT soleus, and 160/6 for HTZ soleus). *P<0.05, significantly different by Student's *t*-test from mean value measured in the WT mice.

Cytosolic calcium concentration ([Ca^2+^]_c_) was determined using the ratiometric Fura-2 calcium probe in mechanically dissected muscle fiber bundles of EDL and soleus muscles from wild-type (WT) and HTZ 4-month-old mice. At this age, no atrophy is evidenced in EDL and soleus muscles from HTZ mice compared to WT [WT EDL: 0.36±0.01 (mean±s.e.m.), HTZ EDL: 0.34±0.02, WT soleus: 0.33±0.01, and HTZ soleus: 0.36±0.02 mg/g body weight; *n*=5 for both muscles]. The resting cytosolic Ca^2+^ level was around 50% higher in EDL muscle fibers from HTZ mice as compared to healthy WT mice (Fig. 1A). This was not observed in soleus muscle in which [Ca^2+^]_c_ values were similar between muscle fibers from HTZ and WT mice (Fig. 1B), demonstrating a specific spatial impairment of the calcium homeostasis.

### Sarcolemmal permeability to calcium (SPCa) increases in EDL from HTZ mice

**Fig. 2.**
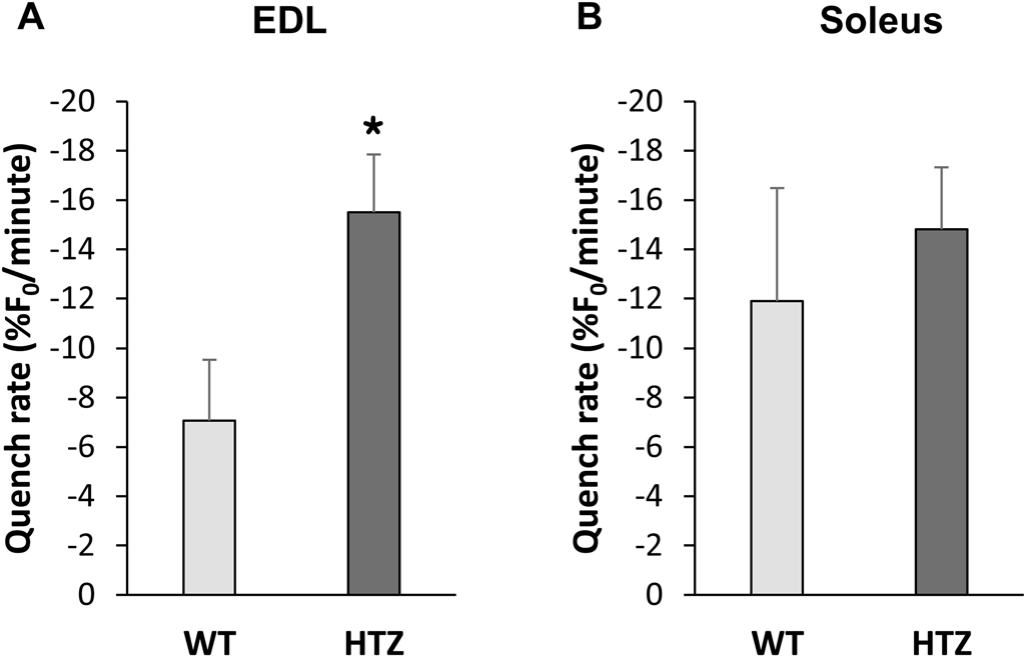
**Sarcolemmal permeability to divalent cations (SPCa) in WT and HTZ EDL and soleus muscles.** The SPCa was quantified by measuring inhibition of the Fura-2-associated fluorescence by manganese. The bars represent mean±s.e.m. calculated in *n* cells from *N* animals (*n*/*N*: 12/2 for WT EDL, 54/5 for HTZ EDL, 35/3 for WT soleus, and 55/5 for HTZ soleus). *P<0.05, significantly different by Student's *t*-test from mean value measured in the WT mice.

In order to assess the possibility that the higher [Ca^2+^]_c_ in HTZ EDL muscle fibers could be related to an increase in Ca^2+^ entrance, we used the manganese (Mn^2+^)-quenching technique. Mn^2+^ enters cells via the same routes as Ca^2+^ but accumulates inside the cell. As Mn^2+^ quenches the fluorescence of Fura-2, the reduction of the intensity of the fluorescent probe is an indicator of the time integral of Ca^2+^ influx (Hopf et al., 1996). The quench rate calculated in HTZ EDL muscle fibers was twice that measured in WT EDL muscle fibers (Fig. 2A). On the other hand, according to the lack of difference in resting [Ca^2+^]_c_, quench rates were found to be similar in soleus muscle fibers from HTZ and WT mice (Fig. 2B). These data implicate increased SPCa in the higher [Ca^2+^]_c_ in a muscle-specific pattern.

### Sarcoplasmic reticulum Ca^2+^ content is higher in EDL from HTZ mice

**Fig. 3.**
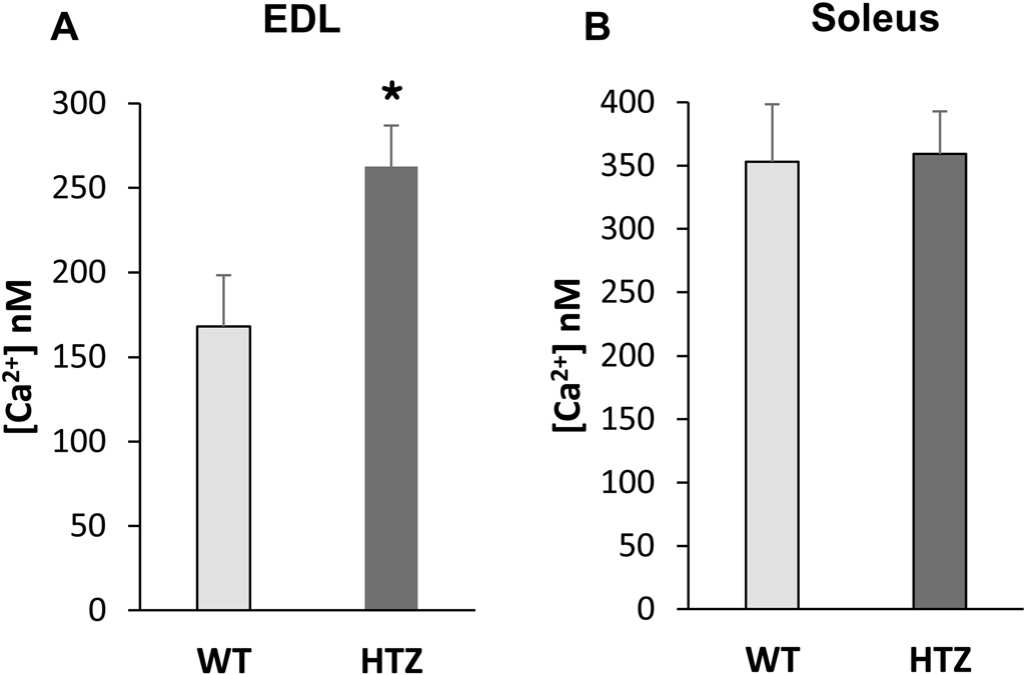
**Calcium releasable from the SR in WT and HTZ EDL and soleus muscles.** Releasable SR [Ca^2+^] was measured in fibers treated with 4-cmc which simultaneously triggers Ca^2+^ release from SR via RYR1 activation and inhibits Ca^2+^ uptake into SR via SERCA inhibition. The bars represent mean [Ca^2+^]_c_±s.e.m. calculated in *n* cells from *N* animals (*n*/*N*: 85/5 for WT EDL, 72/5 for HTZ EDL, 92/5 for WT soleus, and 91/5 for HTZ soleus). *P<0.05, significantly different by Student's *t*-test from mean value measured in the WT mice.

The high resting [Ca^2+^]_c_ observed in the EDL muscle fibers from HTZ mice may also depend on alterations of intracellular calcium storage compartments. Given that sarcoplasmic reticulum (SR) is the main regulatory intracellular compartment of calcium level, we further evaluated the releasable SR calcium content in EDL and soleus muscle fibers from HTZ and WT mice by 4-chloro-*m*-cresol (4-cmc) application. At the concentration used, this compound activates Ca^2+^ release from SR via the ryanodine receptor Ca^2+^ channel and simultaneously blocks Ca^2+^ uptake into SR by inhibiting the sarcoplasmic endoplasmic reticulum Ca^2+^-ATPase (SERCA) Ca^2+^ pump. Consequently, the amplitude of the 4-cmc-induced cytosolic Ca^2+^ rise is dependent on releasable SR Ca^2+^. When exposed to 4-cmc, the increase of [Ca^2+^]_c_ was larger in EDL muscle fibers from HTZ mice as compared to WT ones (Fig. 3A) in agreement with an increased Ca^2+^ content releasable from the SR. Under the same experimental conditions, similar cytosolic calcium elevations were measured in soleus muscle fibers from both genotypes (Fig. 3B), demonstrating an unchanged Ca^2+^ SR content in this muscle.

### Muscle fiber tetanic force is reduced in EDL from HTZ mice

**Fig. 4.**
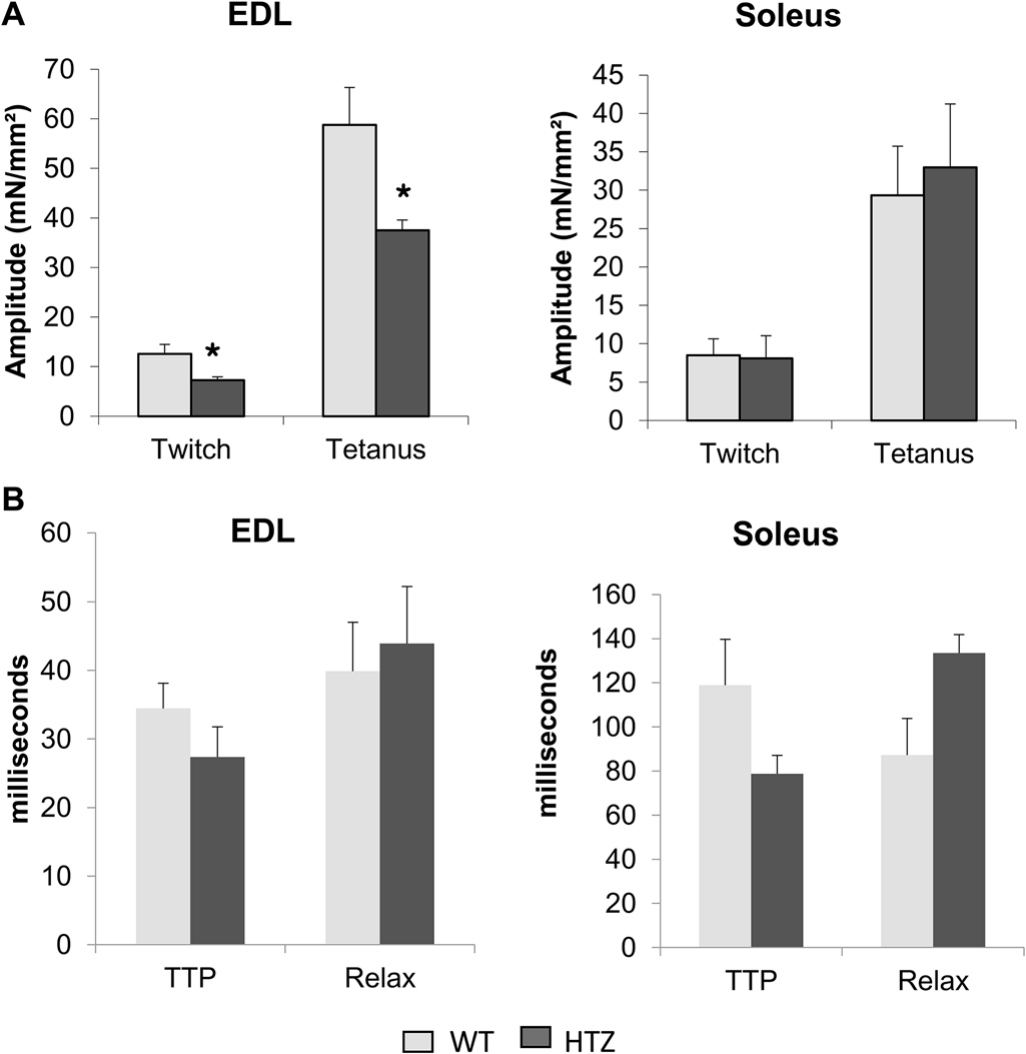
**Force tension development in WT and HTZ EDL and soleus muscles.** (A) Amplitude of electrical-triggered force by pacing designed to induce single twitch or tetanus. (B) Kinetics of the twitch. In A and B, the bars represent mean±s.e.m. measured in *n* cells from *N* animals (*n*/*N*: 8/5 for WT EDL, 7/5 for HTZ EDL, 12/5 for WT soleus, and 10/5 for HTZ soleus). *P<0.05, significantly different by Student's *t*-test from mean value measured in the WT mice. TTP, time to peak; Relax, constant of relaxation.

We previously found that whole-muscle tetanic force was decreased in tibialis anterior muscle from HTZ mice (Durieux et al., 2010b). In the present study, we assessed this alteration at the muscle fiber level in EDL and soleus in order to correlate calcium homeostasis defect and impairment of muscle function. Electrical-triggered force development was acquired after a single twitch or a train of stimulation eliciting perfect tetanus. As reported in Fig. 4A, both twitch and tetanic specific amplitude were decreased in muscle bundles from HTZ EDL as compared to WT ones. This was not found in soleus muscle bundles (Fig. 4A). The time to peak and the constant of relaxation of the twitch were also measured to evaluate contractile responses and were similar in HTZ compared to WT both in EDL and soleus muscles (Fig. 4B).

### Ca^2+^ transient characteristics are unchanged in HTZ EDL and soleus muscles

**Fig. 5.**
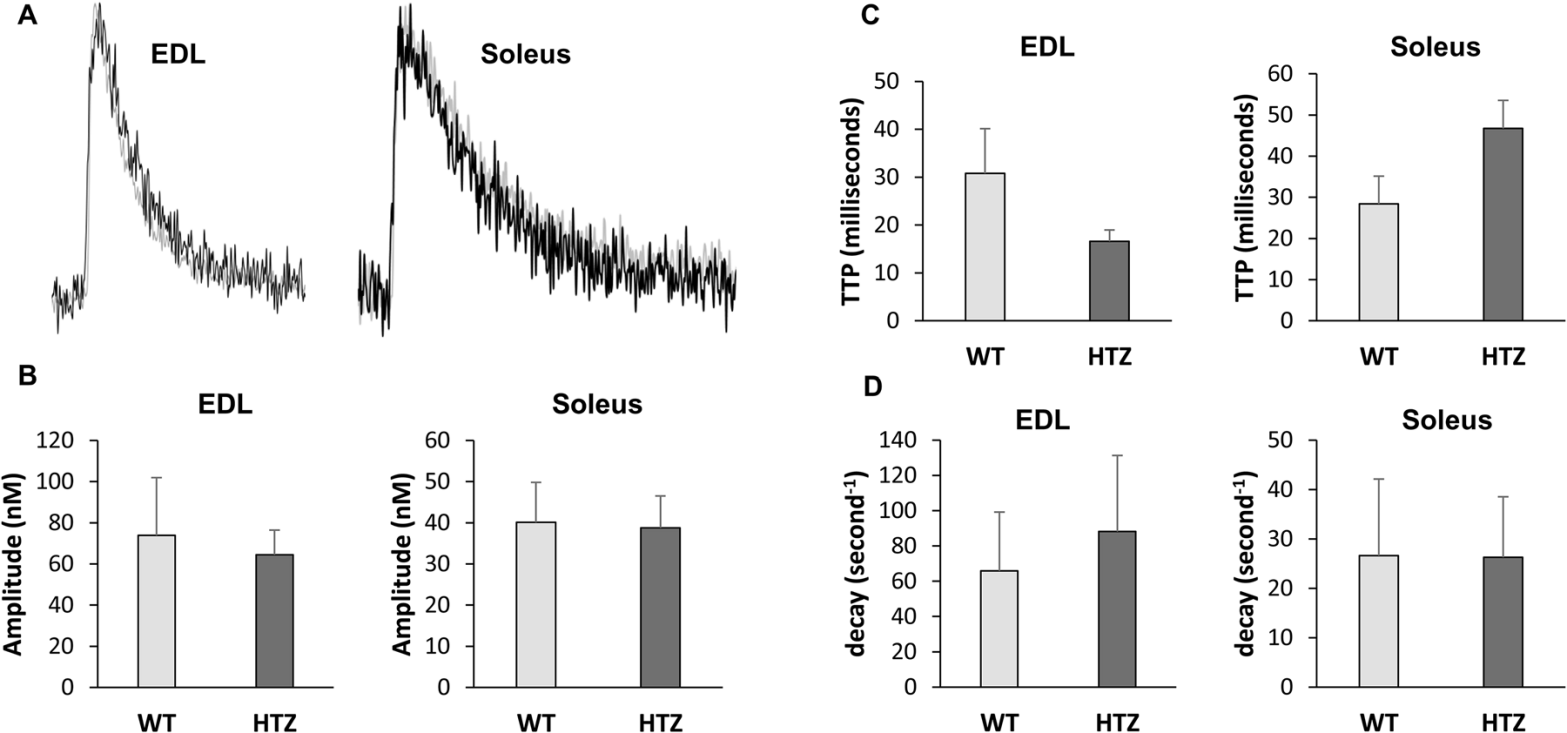
**Characteristics of calcium transient in WT and HTZ EDL and soleus muscles.** (A) Typical Ca^2+^ transient recorded in single EDL and soleus muscle fibers from WT (black) and HTZ (light grey) mice. (B) Amplitude of the calcium transient. (C) Time to peak (TTP). (D) Rate constant of calcium decay. The bars represent mean±s.e.m. value calculated in *n* cells from *N* animals (*n*/*N*: 9/5 for WT EDL, 8/5 for HTZ EDL, 12/5 for WT soleus, and 8/5 for HTZ soleus).

In order to determine whether the lower muscle fiber force development is related to alterations in the excitation-Ca^2+^ release process, we determined the Ca^2+^ transient characteristics in muscle fibers. Ca^2+^ transient (Fig. 5A) is composed of the rapid increase of [Ca^2+^]_c_ induced by electrical stimulation followed by the progressive [Ca^2+^]_c_ decrease linked to Ca^2+^ transport out of the cytoplasm. As illustrated in Fig. 5A, the shapes of calcium transients recorded in the EDL and soleus muscle fibers from WT mice completely overlapped those of calcium transients recorded in HTZ animals. Accordingly, no significant differences were found in the amplitude (Fig. 5B), the time to peak (Fig. 5C) and the rate constant of calcium decay (Fig. 5D) of the Ca^2+^ transient between WT and HTZ fibers from both EDL and soleus muscles.

## DISCUSSION

Understanding underlying pathophysiological mechanisms, especially in animal models, is crucial in designing efficient future therapies, yet still unavailable for *DNM2*-linked CNM patients. We developed a knock-in mouse model of this congenital disease; i.e. the KI-*Dnm2*^R465W^ model, to address this question *in vivo*. We previously identified an alteration of the calcium homeostasis in isolated fibers from FDB muscles in HTZ KI-*Dnm2* mice, which exhibit an increased cytosolic calcium concentration (Durieux et al., 2010b). Here, the calcium homeostasis impairment was better characterized in the fast-twitch EDL and the slow-twitch soleus muscles.

As previously shown in FDB muscle, cytosolic calcium concentration is increased in HTZ EDL muscle. Moreover, our results point toward the plasma membrane as the origin of the defect since membrane permeability to calcium is clearly impaired. Cytosolic calcium concentration is tightly regulated in muscle fibers and the plasma membrane plays an important role in this regulation through integral membrane proteins controlling both calcium entry and efflux. Defective calcium influx or efflux across the plasma membrane has already been shown to be involved in elevated calcium concentration occurring in Duchenne muscular dystrophy resulting from dystrophin deficiency (Deval et al., 2002; Vandebrouck et al., 2002; Zhao et al., 2012). Among the integral membrane proteins, voltage-activated, store-operated, and stretch-regulated channels represent the main ways of entry whereas Na^+^/Ca^2+^-exchanger or Ca^2+^-ATPase pump are involved in calcium extrusion (Vallejo-Illarramendi et al., 2014). The potential implication of one or several of these proteins in the calcium misregulation in our model remains to be determined. However, due to the well-characterized role of the DNM2 in endocytosis, which may be altered by CNM-related mutations (Bitoun et al., 2009; Koutsopoulos et al., 2011), it is tempting to speculate that impairment of endocytosis of calcium channel(s) may increase cell surface channel and calcium entry as already shown for potassium channel (Xu et al., 2009). Strengthening this hypothesis, a dynamin-dependent internalization of the L-type channel Cav1.2 was demonstrated in pancreatic β-cells (Buda et al., 2013) and neurons (Di Biase et al., 2011; Green et al., 2007), and activity of the TRPV4 calcium channel was shown to be regulated by PI4,5P2 and the BAR-domain protein PACSIN3 (Garcia-Elias et al., 2013), both known partners of DNM2 (Klein et al., 1998; Linkermann et al., 2009).

On the other hand, we show that calcium content of the SR is also slightly elevated in HTZ muscles. This suggests that increased cytosolic calcium concentration is not the result of a leaky SR and then reinforces the hypothesis of the primary impact taking place at the plasma membrane. We can hypothesize a regulatory mechanism at the SR in order to compensate the cytosolic increase or an independent unidentified DNM2-linked impairment at the SR membrane leading to increased calcium uptake. Co-localization of DNM2 with the SR SERCA Ca^2+^ pump has already been demonstrated in mouse muscle (Durieux et al., 2010b) and is in agreement with this hypothesis.

Plasma membrane permeability to calcium appears involved in the pathophysiology of the myopathy related to *DNM2* mutation. This differs from the defects of calcium handling already shown in other congenital myopathies (Nance et al., 2012) including disorders linked to *BIN1*, *RYR1* or *MTM1* mutations that are centered on defects of triad membrane components, i.e. T-tubules and SR. In particular, animal models of CNM linked to MTM1 deficiency show disorganization of the T-tubule system, reduction in Ca^2+^ release from the SR, and ECC defect without change in basal cytosolic calcium concentration (Al-Qusairi et al., 2009; Dowling et al., 2009), in agreement with MTM1 and BIN1 expression at the T-tubule system (Buj-Bello et al., 2008; Dowling et al., 2009; Lee et al., 2002; Razzaq et al., 2001; Tjondrokoesoemo et al., 2011). Although affected calcium handling mechanism may differ, altogether these data emphasize calcium homeostasis alterations as possible common pathomechanism in CNM.

The calcium transient is central in the ECC in muscle fibers. It results from the neuronal action potential transmitted to the fibers via the neuromuscular junction, and it triggers sarcomere shortening. Interestingly, we found that amplitude and kinetics characteristics of the calcium transient are normal in HTZ EDL muscles which develop a weaker force. This result suggests that contractile property impairment is probably not directly due to the calcium defect in affected EDL muscle. This apparently diverges from recent report of defective ECC in zebrafish expressing the p.S619L *DNM2* mutant (Gibbs et al., 2014). These discrepancies might be due to the difference in muscles studied or overexpression of a *DNM2* mutant linked to the severe form of the disease in a developing muscle (Gibbs et al., 2014) versus endogenous expression of a *DNM2* mutant linked to a milder form of AD-CNM in an adult muscle (the present study). Characterization of the calcium homeostasis in additional muscles and in animal models expressing additional *DNM2* mutations will be useful to better establish a potential relationship between the type of calcium defect and clinical severity in *DNM2*-related CNM.

By analyzing both EDL and soleus muscles, we were able to correlate the abnormal cytosolic calcium concentration with contraction defect. Indeed, only EDL shows both alterations. One may also hypothesize that a long-term adaptation to increased concentration of calcium, which is a central secondary messenger in cell biology serving a plethora of essential cell functions, may be indirectly involved in muscle weakness. Indeed, important calcium-dependent mechanisms take a central place in muscle homeostasis such as mitochondrial function by regulation of the respiratory chain, gene expression by a variety of calcium-dependent transcription factors, protein function by phosphorylation, or protein degradation through activation of the calpains; a family of non-lysosomal calcium-activated proteases (Gehlert et al., 2015). Future studies should define how the identified impairment of calcium homeostasis may affect these calcium-dependent processes and contribute to the pathomechanisms of the *DNM2*-related CNM.

One central question for the *DNM2*-related diseases is to understand why mutations of the ubiquitously expressed DNM2 lead to a tissue-specific phenotype only affecting skeletal muscles in AD-CNM (Bitoun et al., 2005) or peripheral nervous system in rare form of Charcot–Marie–Tooth neuropathy (Zuchner et al., 2005). Moreover, all the muscles are not similarly affected in AD-CNM since a temporal and spatial muscle involvement has been described in patients (Fischer et al., 2006). In this context, the correlation between calcium defects and muscle weakness is of particular importance. Indeed, these results suggest that calcium homeostasis impairment might be involved in the development of the tissue-specific phenotype in *DNM2*-related CNM and may also participate to the spatial pattern of muscle involvement. Two mechanisms may underlie the uneven spatial involvement in response to *DNM2* mutations as illustrated here through the different impacts on EDL and soleus muscles in HTZ mice. First, *DMN2* mutation may impair cytosolic calcium concentration depending on the type and the amount of channels participating in SPCa initially expressed at the plasma membrane of a given muscle. Accordingly, the sarcolemmal calcium channels TRPC1 and TRPC3, two modulators of SPCa and cytosolic calcium, are differently expressed in EDL and soleus. In this context, it is interesting to note that TRPV4, known to be regulated by DNM2, may form a heteromeric channel with TRPC1 (Fraysse et al., 2003; Garcia-Elias et al., 2013; Rosenberg et al., 2004; Woo et al., 2014; Zhang et al., 2016). The second mechanism may be related to the different calcium handling mechanisms occurring in fast- and slow-twitch muscles fibers in relation to their contractile phenotype (Dowling et al., 2016; Schiaffino and Reggiani, 2011). In particular, EDL and soleus muscles differ in regard to expression of specific SR Ca^2+^-ATPase SERCA isoforms, mitochondrial content, and development of SR, the main intracellular calcium compartment. Resulting from these differences, we found that [Ca2+]_c_ is significantly higher in soleus than in EDL (ANOVA and Bonferroni post-hoc test, *P<0.05*) as previously reported (Carroll et al., 1999; Fraysse et al., 2003, 2006; Gailly et al., 1993). A similar trend is observed for SPCa, although not significant. One may hypothesize that soleus muscle is less sensitive to *DNM2* mutations due to its naturally higher SPCa and cytosolic calcium content. Analysis of calcium homeostasis in additional affected or unaffected muscles will be necessary to assess these hypotheses that will be essential to evaluate efficiency of future experimental therapeutic strategies developed to counteract this particular deficit.

In conclusion, we show that the elevated cytosolic Ca^2+^ concentration in EDL muscles from a KI mouse model of *DNM2*-linked CNM is associated with an increased sarcolemma permeability to Ca^2+^. Our results suggest that calcium defect is probably not a primary cause of decreased force generation by compromised sarcomere shortening but may be involved in long-term deleterious consequences on muscle physiology which remain to be determined. In addition, we better characterize here the spatial pattern of muscle involvement in KI-*Dnm2* mice by demonstrating that functional impairment also affects the EDL muscle in absence of atrophy. Importantly, by correlating calcium homeostasis impairment and contractile properties defect, this study highlights the first pathomechanism which may trigger the spatial muscle involvement occurring in the *DNM2*-related CNM. Although molecular changes underlying calcium defects need to be further characterized, our results open the way toward development of a therapeutic approach devoted to normalizing calcium content in *DNM2*-linked CNM.

## MATERIALS AND METHODS

### Dissection of native muscle fibers

The dynamin 2 mutant C57BL/6 mouse line was established by homologous recombination using standard techniques (Durieux et al., 2010b). Animal studies were performed in compliance with the French animal welfare laws, guidelines and policies. Extensor digitorum longus (EDL) and soleus muscles were removed from male mice of 4 months of age under deep urethane anesthesia (1.2 g/kg body weight). Soon after, the mice, still anaesthetized, were euthanized by anesthetic overdose. Muscles were pinned in SYLGARD-coated dishes containing normal physiological solution (NPS) composed of 148 mM NaCl, 4.5 mM KCl, 2.5 mM CaCl_2_, 1 mM MgCl_2_, 10 mM HEPES (4-(2-hydroxyethyl)-1-piperazineethanesulfonic acid), and 5.5 mM glucose, pH 7.4, at room temperature. All the chemicals were from Sigma-Aldrich (France). Small bundles of 10-15 fibers arranged in a single layer were dissected lengthwise, from tendon to tendon, using microscissors. Muscle preparations were loaded during 1 h with 3 µM of the Fura-2 AM fluorescent calcium probe (Thermo Fisher Scientific, France).

### Force tension development

After Fura-2 loading, muscle fascicles were mounted in an experimental chamber allowing measurement of isometric force and cytosolic calcium concentration, at rest or under electrical stimulation. One extremity was attached to a hook linked to a micromanipulator and the other extremity was fixed to an electromagnetic force-transducer device described elsewhere (Blanc et al., 1999). Muscle preparations were stretched until the twitch amplitude elicited by electrical stimuli (5 ms duration, 14 V, 0.2 Hz) reached a maximum. The diameter of each contracting fiber was measured to calculate their area. Sum of the areas was used to calculate the cross-sectional area (mm²) of the whole muscle bundle and to normalize force amplitude (mN). Electrical-triggered force development was acquired under two stimulus protocols: (1) 5 ms duration/14 V/0.2 Hz protocol designed to elicit single separated twitch; (2) 20 pulses of 5 ms duration/14 V/100 Hz protocol designed to elicit perfect tetanus. The evaluation of the contractile responses was done by measuring the amplitude (mN/mm²), the time to peak (ms) and the time constant of relaxation (s^−1^) of the twitch, and the amplitude (mN/mm²) of the tetanus. All the experiments were conducted at room temperature.

### Resting, transient and releasable SR calcium

Ratiometric Fura-2 fluorescence measurements were made using optical excitation filters of 380 and 360 nm and an IonOptix microStepper Switch integrated system (IonOptix, Ireland). Emitted fluorescence (510 nm) was background subtracted. Cytosolic calcium concentration ([Ca^2+^]_c_) was calculated at rest according to a modified method from Grynkiewicz and collaborators (Fraysse et al., 2006; Grynkiewicz et al., 1985). A pseudo-ratiometric approach was used to acquire calcium transients. Preparations were electrically stimulated with a pace protocol (0.5 Hz, 4 ms duration, 14 V) designed to induce a single action potential. Fura-2 fluorescence was first recorded at 1000 Hz under 380 nm excitation during 10 electrical stimulations. Thereafter, Fura-2 fluorescence was recorded at 360 nm. The 10 records were averaged at each excitation wavelength. Finally the calcium transient was calculated by making the ratio of the means (360/380) and transformed in [Ca^2+^] values using the method described for resting [Ca^2+^]_c_ determination. The time to peak, the amplitude and the rate constant of calcium decay (by fitting a monoexponential on the recovery phase of the calcium transient) were calculated.

Releasable SR Ca^2+^ assessment was achieved by application of 1 mM 4-chloro-*m*-cresol (4-cmc) which activates the ryanodine receptor Ca^2+^ channel and inhibits the sarcoplasmic endoplasmic reticulum Ca^2+^-ATPase Ca^2+^ (SERCA) pump. When exposed to 4-cmc, muscle fiber [Ca^2+^]_c_ increases reaching a plateau after few minutes. 4-cmc was applied after a train of electrical pulses (0.5 Hz, 4 ms duration, 14 V for 30 s) to normalize SR Ca^2+^ content between muscle preparations.

### Determination of sarcolemmal permeability to divalent cations

The manganese quench technique was used to determine the sarcolemmal permeability to divalent cations (SPCa). Muscle preparations were first perfused for 2 min with NPS containing 0.5 mM Mn^2+^ as a surrogate of Ca^2+^ (quenching solution). Then, the quenching solution was applied to muscle fibers for 2-4 min. During the whole quenching protocol the fluorescence of Fura-2 excited at 360 nm was acquired at 1 Hz. The quench rates were determined using linear regression analysis of fluorescence signal and expressed as the decline per minute of the initial fluorescence intensity.

### Statistical analysis

The data were analyzed using Microsoft Excel and were compared using two-tailed unpaired Student's *t*-test. *P*-values less than 0.05 were considered significant.
